# Kinetics-based development of two-stage continuous fermentation of 1,3-propanediol from crude glycerol by *Clostridium butyricum*

**DOI:** 10.1186/s13068-024-02486-5

**Published:** 2024-03-07

**Authors:** Xiao-Li Wang, Ya-Qin Sun, Duo-Tao Pan, Zhi-Long Xiu

**Affiliations:** 1 MOE Key Laboratory of Bio-Intelligent Manufacturing, School of Bioengineering, Dalian University of Technology, No. 2 Linggong Road, Ganjingzi District, Dalian, 116024 Liaoning People’s Republic of China; 2 Institute of Information and Engineering, Shenyang University of Chemical Technology, Shenyang, 110142 Liaoning People’s Republic of China

**Keywords:** 1,3-Propanediol, Crude glycerol, Continuous fermentation, Kinetic model, *Clostridium butyricum*

## Abstract

**Background:**

Glycerol, as a by-product, mainly derives from the conversion of many crops to biodiesel, ethanol, and fatty ester. Its bioconversion to 1,3-propanediol (1,3-PDO) is an environmentally friendly method. Continuous fermentation has many striking merits over fed-batch and batch fermentation, such as high product concentration with easy feeding operation, long-term high productivity without frequent seed culture, and energy-intensive sterilization. However, it is usually difficult to harvest high product concentrations.

**Results:**

In this study, a three-stage continuous fermentation was firstly designed to produce 1,3-PDO from crude glycerol by *Clostridium butyricum*, in which the first stage fermentation was responsible for providing the excellent cells in a robust growth state, the second stage focused on promoting 1,3-PDO production, and the third stage aimed to further boost the 1,3-PDO concentration and reduce the residual glycerol concentration as much as possible. Through the three-stage continuous fermentation, 80.05 g/L 1,3-PDO as the maximum concentration was produced while maintaining residual glycerol of 5.87 g/L, achieving a yield of 0.48 g/g and a productivity of 3.67 g/(L·h). Based on the 14 sets of experimental data from the first stage, a kinetic model was developed to describe the intricate relationships among the concentrations of 1,3-PDO, substrate, biomass, and butyrate. Subsequently, this kinetic model was used to optimize and predict the highest 1,3-PDO productivity of 11.26 g/(L·h) in the first stage fermentation, while the glycerol feeding concentration and dilution rate were determined to be 92 g/L and 0.341 h^−1^, separately. Additionally, to achieve a target 1,3-PDO production of 80 g/L without the third stage fermentation, the predicted minimum volume ratio of the second fermenter to the first one was 11.9. The kinetics-based two-stage continuous fermentation was experimentally verified well with the predicted results.

**Conclusion:**

A novel three-stage continuous fermentation and a kinetic model were reported. Then a simpler two-stage continuous fermentation was developed based on the optimization of the kinetic model. This kinetics-based development of two-stage continuous fermentation could achieve high-level production of 1,3-PDO. Meanwhile, it provides a reference for other bio-chemicals production by applying kinetics to optimize multi-stage continuous fermentation.

**Graphical Abstract:**

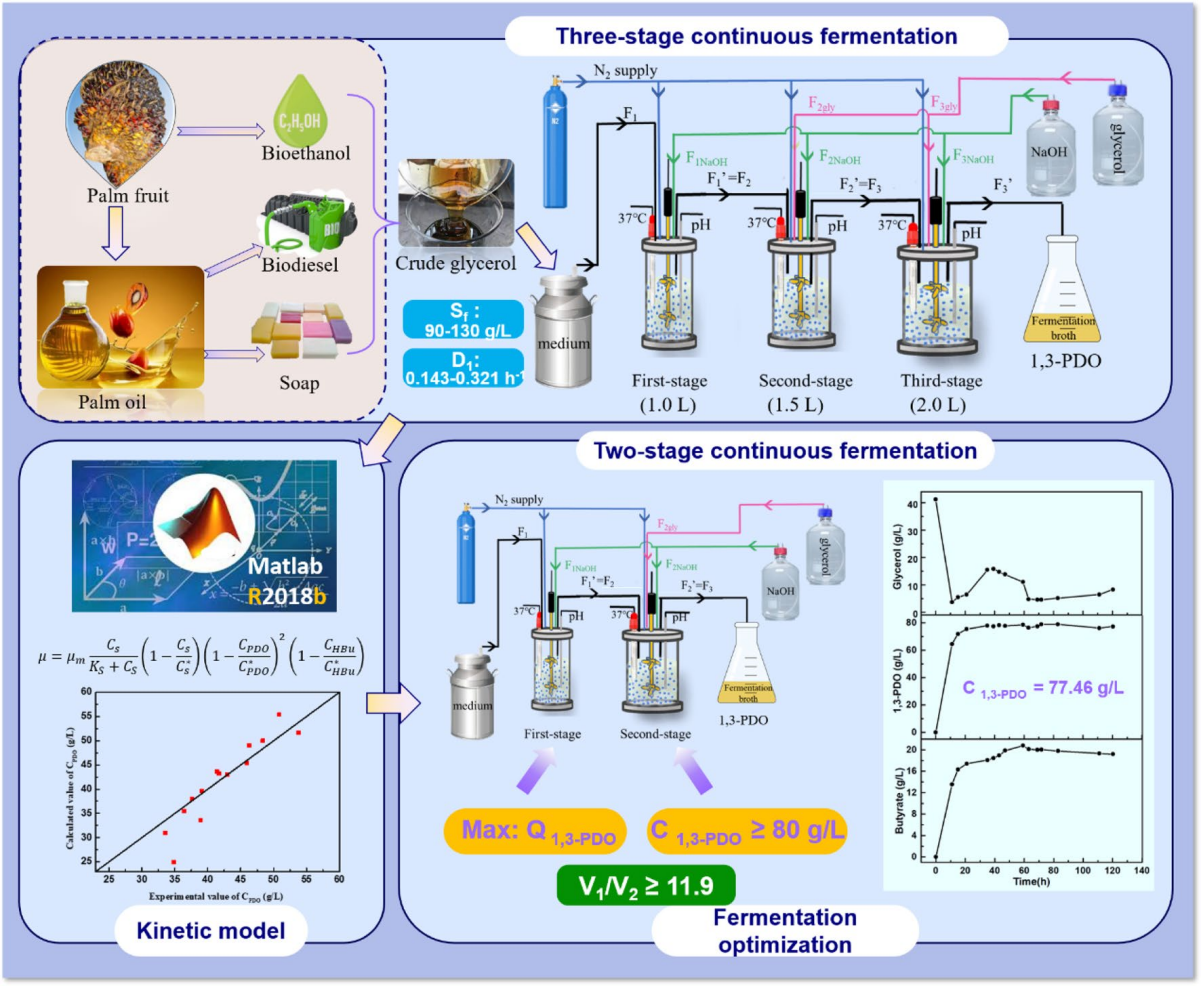

## Background

Crude glycerol is produced as the by-product of biodiesel, soap, fatty acid, fatty ester, and ethanol industries from the conversion of many agricultural crops such as corn, colza, palm, and olive [1, 2]. The rapid production of biodiesel results in a large surplus of waste crude glycerol, which not only fails to maximize the crop industry economy, but also aggravates environmental pollution due to the disposal of crude glycerol [3]. Therefore, this circumstance has stimulated researchers to investigate how to transform this low-value glycerol to valuable products. 1,3-Propanediol (1,3-PDO), a valuable product derived from glycerol, has been broadly applied in solvents, cosmetics, medicine, and plastics [4]. Especially, it can be utilized to synthesize polyester and polyurethane [5]. For instance, polytrimethylene terephthalate (PTT), an excellent polyester material, has potential market prospects in plastic and textile industry owing to its superior resilience and stretching characteristics [6, 7].

1,3-PDO can be synthesized by microbial fermentation and chemical synthesis. Recently, chemical process has restricted the 1,3-PDO production in large-scale because of its harsh reaction conditions and the growing public concerns about sustainable development [8, 9]. By contrast, the fermentation engineering, as a green and sustainable process, can be performed under a milder and more environment-friendly condition [10]. There are many reported wild-type strains that could transform glycerol to 1,3-PDO by micro-aerobic or anaerobic fermentation, including the genus *Clostridium*, *Klebsiella*, *Enterobacter*, *Lactobacillus*, and *Citrobacter*, and so on [11–15]. *Clostridum butyricum*, a probiotic and B_12_-independent bacterium, has been well studied for 1,3-PDO production in batch [16], repeated batch [17, 18], fed-batch [19], repeated fed-batch [8], sequential fed-batch [20], continuous [21] and two-stage continuous fermentations [22]. However, in batch and continuous fermentation, the lower 1,3-PDO concentrations are not suitable for scale-up production. In fed-batch, repeated fed-batch, and sequential fed-batch fermentations, although a satisfactory product concentration can be obtained, it is produced with a complicated feed control strategy in fermentation process, such as pulsed, continuous, and pulsed continuous feeding [23, 24]. Continuous fermentations have many striking advantages over batch and fed-batch processes, such as more economical, long-term high productivity without frequent seed culture and energy-intensive sterilization. Of course, it does have serious weaknesses including low product concentration, long-term sterility maintenance. Thus, the stable and automatic continuous fermentation has attracted the interest of researchers to investigate how to promote high concentrations of 1,3-PDO while achieving high productivity, which would further promote the industrial production of 1,3-PDO.

During fermentation process, glycerol concentration and the accumulation of product and by-products have negative effects due to substrate inhibition, osmotic stress, oxidative stress, and toxic stress on 1,3-PDO production and microbial growth [16, 25]. Unfortunately, the by-products are always accompanied by 1,3-PDO formation because of the intracellular metabolic balance between the oxidative and reductive pathways. Many studies have focused on reducing by-products and applying appropriate substrate concentrations to stimulate 1,3-PDO production. Continuous feeding, one-pulse and two-pulse feeding strategies were adopted to ensure different glycerol concentrations for a better fermentation performance [26]. The distinct effects of butyrate and acetate on 1,3-PDO production and bacterial growth have highlighted the metabolic flexibility of *C. butyricum* [27]. However, most of optimized processes are based on numerous experiments and a series of in-depth optimization design.

In continuous microbial fermentation, the initial substrate concentration and dilution rate were usually set based on some experiences and reported values. However, if the optimal operation conditions are needed for production, it is impossible that many experiments are conducted for optimal parameters considering the large workload and the intricate relationship among all products, substrate, and microbial growth. Thus, the kinetic model was proposed as an excellent auxiliary tool that employs mathematical language for the analysis of microbial fermentation processes. Many reports have proposed various kinetic models to describe the relationship among substrates, products, and microbial growth based on different 1,3-PDO producers. Zeng et al. established an inhibition model which focuses on the substrate and products inhibition on the growth of *K. pneumoniae* and *C. butyricum* [28]. As for *C. butyricum*, some reports investigated the effects of different culture conditions (initial glycerol concentrations, yeast extract concentrations, temperature, and pH) on the kinetic behavior of 1,3-PDO production [29]. A detailed model had been proposed to describe 1,3-PDO, by-products, biomass, and substrate concentration in batch fermentation and its modified model could be employed to predict fed-batch fermentation [19].

This study is supposed to investigate the three-stage continuous fermentation, which was performed at different glycerol feeding concentration and dilution rates, to achieve high concentrations and yields of 1,3-PDO and ensure a low level of remaining glycerol (< 7 g/L). Furthermore, a novel kinetic model should be established based on the collected experimental data and serve as a useful tool for optimizing and guiding fermentation process. Finally, a two-stage continuous fermentation would be designed based on the developed kinetic model.

## Results and discussion

### Coordinated production of 1,3-PDO in three-stage continuous fermentations

#### Robust cell growth in the first stage fermentation

The first stage fermentation would play an important role for providing seed for the other two fermenters. The good robustness was expected for cell growth of *C. butyricum* DL07. Our previous work showed that this strain exhibited high tolerance to crude glycerol in three-pulsed fed-batch fermentation [20]. Thus, high glycerol concentrations (90–130 g/L) were selected in feeding medium in the first stage fermentation at various dilution rates. As shown in Table 1, the dilution rate of feeding medium $\left( {D_{1} } \right)$ varied from 0.143 to 0.321 h^−1^ to avoid metabolism changes of the strain resulting from excessive or deficient glycerol. Indeed, the actual dilution rates of fermentation $\left( {D^{\prime}_{1} } \right)$ are greater than the set dilution rates of feeding media on account of the addition of NaOH solution for adjusting pH during the fermentation. In the first stage fermentation, the maximum biomass productivity of 1.24 g/(L·h) was achieved at 90 g/L feeding glycerol with a high feeding rate of 0.321 h^−1^, accompanied by the highest 1,3-PDO productivity (11.77 g/(L·h)). While feeding glycerol concentration increased to 130 g/L, the maximum 1,3-PDO concentration reached 53.82 g/L at a feeding dilution rate of 0.143 h^−1^, accompanied by a lower productivity of 8.29 g/(L·h). Moreover, the lowest biomass productivity of 0.66 g/(L·h) was obtained, and butyrate (12.39 g/L) was accumulated substantially. Considering the lowest productivities of biomass with the high residual glycerol of 17.91 g/L based on a low feeding dilution rate (0.143 h^−1^) at 130 g/L glycerol, so no lower feeding dilution rate was explored. As for by-products, when 90 g/L glycerol in the feeding medium was pumped into the first stage fermentation accompanied by the highest feeding rate of 0.321 h^−1^, the ratio of butyrate to acetate in fermentation broth was 1.13. However, since the feeding glycerol concentration increased to 130 g/L at the lowest dilution rate of 0.143 h^−1^, the resulting ratio of butyrate to acetate was dramatically increased up to 3.20 (Table 4).

**Table 1:** The experimental results in the first stage continuous fermentations

The first stage continuous fermentation										
*S*_f_	$D_{1}$	$D^{\prime}_{1}$	$C_{{1\;{\text{S}}}}$	Biomass	1,3-PDO	Butyrate	Acetate	Lactate	$Q_{{1_{{{\text{Biomass}}}} }}$	$Q_{{1_{{{\text{PDO}}}} }}$
(g/L)	(h^−1^)		(g/L)						(g/(L·h))	
90	0.222	0.237	5.81 ± 1.19	4.17 ± 0.17	42.94 ± 0.71	6.65 ± 0.18	4.83 ± 0.08	1.67 ± 0.04	0.99	10.18
	0.256	0.269	13.43 ± 1.46	4.04 ± 0.13	38.89 ± 0.70	6.35 ± 0.34	4.32 ± 0.24	2.18 ± 0.31	1.09	10.46
	0.279	0.294	15.38 ± 1.18	3.75 ± 0.16	37.59 ± 0.95	5.58 ± 0.24	4.88 ± 0.12	1.76 ± 0.07	1.10	11.05
	0.321	0.338	21.69 ± 2.17	3.66 ± 0.15	34.82 ± 1.06	5.06 ± 0.24	4.49 ± 0.13	1.67 ± 0.07	1.24	11.77
100	0.200	0.216	4.60 ± 1.22	4.15 ± 0.10	45.93 ± 0.27	7.50 ± 0.84	4.98 ± 0.77	1.30 ± 0.13	0.90	9.83
	0.211	0.230	14.22 ± 0.58	4.04 ± 0.17	41.73 ± 0.87	6.64 ± 0.22	5.77 ± 0.09	0.98 ± 0.03	0.93	9.43
	0.232	0.247	19.86 ± 1.01	3.66 ± 0.15	39.06 ± 0.52	5.95 ± 0.09	5.48 ± 0.07	0.93 ± 0.03	0.90	9.65
	0.253	0.268	25.80 ± 1.01	3.49 ± 0.01	36.41 ± 0.50	5.34 ± 0.08	5.02 ± 0.08	0.92 ± 0.02	0.94	9.76
	0.274	0.289	32.16 ± 2.11	3.16 ± 0.06	33.57 ± 0.18	4.71 ± 0.09	4.61 ± 0.09	0.74 ± 0.05	0.91	9.70
110	0.148	0.160	3.29 ± 0.60	4.25 ± 0.21	50.85 ± 0.45	9.59 ± 0.23	5.67 ± 0.17	2.34 ± 0.04	0.68	8.14
	0.167	0.180	7.29 ± 5.71	4.82 ± 0.37	48.37 ± 2.52	9.95 ± 2.30	4.20 ± 1.43	2.40 ± 0.38	0.87	8.71
	0.173	0.185	14.38 ± 2.16	3.73 ± 0.21	46.30 ± 0.86	7.30 ± 0.44	6.59 ± 0.24	1.65 ± 0.17	0.69	8.56
	0.200	0.212	26.17 ± 1.88	3.40 ± 0.09	41.42 ± 0.83	6.27 ± 0.15	5.85 ± 0.25	1.36 ± 0.07	0.72	8.78
130	0.143	0.154	17.91 ± 1.43	4.26 ± 0.27	53.82 ± 0.71	12.39 ± 0.72	3.87 ± 0.34	2.25 ± 0.35	0.66	8.29

*D*_1_: feeding rates of medium; $D^{\prime}_{1}$: dilution rates; *S*_f_: initial glycerol concentration in feeding medium; *C*_1 s_: residual glycerol concentration in the reactor; *Q*_Biomass_: productivity of biomass; *Q*_PDO_: productivity of 1,3-PDO

In continuous fermentations, *C. butyricum* DSM 5431 subjected to cell recycling could achieved 1,3-PDO productivity of 13.3 g/(L·h) with a feeding glycerol concentration of 26.5 g/L, which is the highest reported value [31]. Beyond that, the next highest 1,3-PDO productivity was 10.3 g/(L·h) by *C. butyricum* VPI 3266 in one-stage continuous fermentation, but only 30.0 g/L 1,3-PDO was produced [32]. Microbial consortium C2-2M could achieve 57.86 g/L 1,3-PDO in one-stage continuous fermentation, which is the best result for concentration so far [21]. *C. butyricum* DL07, used in this study, had been reported to achieve 104.8 g/L 1,3-PDO in fed-batch fermentation [20]. Therefore, a three-stage continuous fermentation was investigated for a high 1,3-PDO concentration and productivity. In the first stage continuous fermentation, the 1,3-PDO concentration increased with decreasing dilution rate under excess glycerol concentrations. After all, the lower dilution rate means that the strain has longer fermentation time to accumulate more 1,3-PDO before being eluted from the bioreactor in continuous fermentations. However, the productivities of 1,3-PDO and biomass showed irregular changes. The cell growth during continuous fermentation is regulated by the concentrations of 1,3-PDO, other by-products and glycerol [33, 34]. These will be validated in the kinetic model established in “Kinetic models development for two-stage continuous 1,3-PDO production” section. Overall, the high cell productivity indicated that microbial growth was at the exponential growth phase. Due to the growth-related and accumulation-suppressed production of 1,3-PDO [35], fluctuations in 1,3-PDO concentrations were observed. In the first stage continuous fermentation, the highest 1,3-PDO productivity reached up to 11.77 g/(L·h). To our knowledge, this 1,3-PDO productivity is among the best results published so far for one-stage continuous fermentation. In addition, 1,3-PDO concentration achieved the best value (53.82 g/L) at 130 g/L glycerol with a lower feeding dilution rate (0.143 h^−1^), but glycerol concentration remained at a high level (17.91 g/L) as well as butyrate concentration. The ratio of butyrate to acetate was up to 3.20, which is more than twice that obtained at 90 g/L feeding glycerol and feeding rate of 0.321 h^−1^, but there was little difference in the residual glycerol concentration (17.91 vs. 21.69 g/L) between the two experimental groups. This result was also presented in continuous fermentation by *C. butyricum* VPI 3266, where the ratio of butyrate to acetate increased since the feeding glycerol concentration rose from 30 to 60 g/L [32]. According to kinetic analysis of *C. butyricum*, the production of butyrate can supply more ATP than acetate for microbial growth and maintenance [36]. However, more NADH can be generated during the production of acetate. Therefore, if acetate is the only by-product, the theoretical maximum yield of 1,3-PDO will be 0.72 mol/mol [36, 37]. Therefore, it is possible that the osmotic stress on the cells caused by accumulation of 1,3-PDO forces the strain to provide more energy (ATP) for cell survival, leading to more production of butyrate.

#### Stable 1,3-PDO production in the second stage continuous fermentation

A sequential fed-batch fermentation has been successfully applied in *C. butyricum* DL07 for at least eight cycles, indicating that the maintenance of a robust seed state in the exponential phase of cell growth can initiate a new fermentation [4, 20]. Similarly, in the first stage fermentation, the strain was in a relatively robust growth state due to sufficient nutrition and weak inhibition from product and substrate. Thus, a three-stage continuous fermentation was explored, aiming to enhance 1,3-PDO production during second stage fermentation. Typically, the concentration of glycerol is maintained at 20 g/L to ensure 1,3-PDO production during fed-batch fermentation process [20, 39]. Therefore, in two-stage continuous fermentation, cells from the first stage fermentation proceed with the second stage fermentation process with the constant addition of crude glycerol for maintaining the approximate glycerol concentration of 20 g/L (13–24 g/L). The glycerol would be automatically and continuously fed into the bioreactor according to the relationship between glycerol consumption and the addition of 5 M NaOH solution [20]. As a result, during the second stage fermentation, the concentrations of 1,3-PDO reached 57.79–75.13 g/L with the productivities of 2.77–6.37 g/(L·h) (Table 2). Compared to the first stage fermentation, the 1,3-PDO concentrations increased by 19–23 g/L in the second stage fermentation. Specifically, when 110 g/L glycerol with a feeding rate of 0.148 h^−1^ was added to the first stage fermentation, the lowest 1,3-PDO increase of 18.46 g/L was achieved. The largest increase in 1,3-PDO concentration was 22.97 g/L in the second stage, since the glycerol concentration of feeding medium and the feeding dilution rate were 90 g/L and 0.321 h^−1^, separately. Although the 1,3-PDO productivity gradually increased with the increase in dilution rate, it remained lower than that of its corresponding first stage fermentation. Moreover, the biomass in the second stage fermentation (4.60–6.24 g/L) increased compared to the first stage fermentation. However, the biomass productivity was only 0.07–0.44 g/(L·h), indicating a significant decrease compared to the previous stage of fermentation. Consequently, the concentration of all by-products (butyrate, acetate and lactate) in the second stage fermentation increased to some extent. The data were divided into four groups according to different feeding glycerol concentration (such as 90, 100, 110 and 130 g/L) in the first stage fermentation. As the dilution rate decreased, the accumulated concentrations of butyrate and lactate in the second stage fermentation tended to gradually increase in every group, while the production of acetate was irregular.

**Table 2:** The experimental results in the second stage continuous fermentations

First stage		The second stage continuous fermentation								
*S*_f_	$D_{1}$	$D^{\prime}_{2}$	$C_{{2\;{\text{S}}}}$	Biomass	1,3-PDO	Butyrate	Acetate	Lactate	$Q_{{2_{{{\text{Biomass}}}} }}$	$Q_{{2_{{{\text{PDO}}}} }}$
(g/L)	(h^−1^)	(h^−1^)	(g/L)						(g/(L·h))	
90	0.222	0.174	16.72 ± 0.95	5.21 ± 0.14	65.16 ± 0.54	13.08 ± 0.19	5.38 ± 0.10	3.08 ± 0.25	0.25	4.57
	0.256	0.199	23.62 ± 1.75	5.13 ± 0.19	61.54 ± 0.97	11.80 ± 0.37	5.36 ± 0.24	2.74 ± 0.28	0.30	5.25
	0.279	0.216	16.98 ± 1.43	5.37 ± 0.17	60.28 ± 0.84	11.34 ± 0.18	6.04 ± 0.21	2.64 ± 0.22	0.42	5.67
	0.321	0.246	18.32 ± 1.77	5.16 ± 0.21	57.79 ± 1.22	9.76 ± 0.27	6.52 ± 0.14	2.11 ± 0.08	0.44	6.37
110	0.148	0.119	13.40 ± 1.76	4.85 ± 0.24	69.31 ± 0.83	16.04 ± 0.23	6.15 ± 0.23	4.83 ± 1.02	0.12	2.83
	0.167	0.133	10.41 ± 3.06	6.24 ± 0.28	68.95 ± 1.57	15.09 ± 1.85	4.71 ± 0.39	2.53 ± 0.37	0.25	3.36
	0.173	0.135	20.59 ± 1.47	4.82 ± 2.56	67.17 ± 0.57	13.99 ± 0.36	7.26 ± 0.24	2.28 ± 0.15	0.19	3.38
	0.200	0.152	15.01 ± 1.03	4.60 ± 0.15	64.36 ± 0.90	12.34 ± 0.46	7.04 ± 0.19	1.57 ± 0.14	0.22	3.93
130	0.143	0.110	14.48 ± 0.37	4.60 ± 0.10	75.13 ± 0.73	18.36 ± 0.75	4.76 ± 0.58	2.98 ± 0.47	0.07	2.77

Two-stage continuous fermentation has been reported in *C. butyricum* and *Citrobacter freundii* for further increasing 1,3-PDO concentration, where 1,3-PDO concentrations could reach 46.3 and 41.5 g/L, respectively [22, 38]. Similarly, in this study, stable 1,3-PDO production was achieved in the second stage fermentation by *C. butyricum* DL07, indicating sustained performance of this strain during first stage fermentation. However, a higher 1,3-PDO concentration in the first stage fermentation (Table 1) resulted in a lower 1,3-PDO increase in the second stage fermentation, probably due to an inhibitory effect of high 1,3-PDO concentrations on cells. Notably, the maximum 1,3-PDO concentration was 75.13 g/L in the second stage, which was 29.8% higher than the maximum 1,3-PDO concentration reported in one-stage continuous fermentation by microbial consortium C2-2M [21], and even comparable to that of fed-batch fermentation. However, during the second stage fermentation, 1,3-PDO productivity showed a significant decrease, attributable to the inhibitory effects of all products on bacteria. Although the biomass in the second stage was higher than that in the first one, a significant reduce in the biomass productivity was observed. The high concentrations of main products and by-products including 1,3-PDO, butyrate, and acetate could bring inhibitory effects on the growth of *C. butyricum* and 1,3-PDO formation [27, 32]. Therefore, in the second stage fermentation, cells entered the stationary phase with the highest biomass. In addition, as compared to the first stage fermentation, the ratio of butyrate to acetate increased in the second stage fermentation (Table 4), indicating that the strains would produce more butyrate when suffered from more severe inhibition.

#### The consumption of glycerol in the third stage fermentation

Given the steady progress observed in the second stage fermentation, therefore, the third stage fermentation was proceeded to obtain the highest 1,3-PDO concentration while maintaining a lower residual glycerol concentration. To remain a lower residual glycerol concentration, crude glycerol at different feeding rates were introduced into the third stage fermentation until the residual glycerol concentration dropped below 7 g/L. The 1,3-PDO concentrations exhibited increments, albeit with significantly lower productivities compared to the second stage, particularly in groups with relatively high 1,3-PDO concentrations (Table 3). In all groups of the third stage fermentation, the concentrations of 1,3-PDO exceeded 70.00 g/L. Notably, 80.50 g/L 1,3-PDO as the best result, was harvested in the third stage fermentation, accompanied by a productivity of 1.33 g/(L·h). However, a decrease in biomass occurred in the third stage fermentation compared to the second stage fermentation. Moreover, for most groups, the biomass during the third stage fermentation was higher than that of the first stage fermentation. However, when the dilution rates of the third stage fermentation were low (0.083–0.090 h^−1^), accompanied by high 1,3-PDO concentrations (> 74.00 g/L), the biomass in the third stage fermentation dropped at lowest level in the three-stage fermentation. Obviously, in the third stage fermentation, the ratio of butyrate to acetate increased as the dilution rate decreased. Meanwhile, compared with the first and second stage fermentation, this ratio reached up to the maximum value in the third stage fermentation (Table 4). Similar to the second stage fermentation, the accumulated concentrations of butyrate and lactate in each group gradually increased with decreased dilution rate in the third stage fermentation, while acetate concentrations still exhibited irregular changes.

**Table 3:** The experimental results in the third stage continuous fermentations

First stage		Second stage	The third stage continuous fermentation								
*S*_f_	$D_{1}$	$D^{\prime}_{2}$	$D^{\prime}_{3}$	$C_{{3\;{\text{S}}}}$	Biomass	1,3-PDO	Butyrate	Acetate	Lactate	$Q_{{3_{{{\text{Biomass}}}} }}$	$Q_{{3_{{{\text{PDO}}}} }}$
(g/L)	(h^−1^)	(h^−1^)	(h^−1^)	(g/L)						(g/(L·h))	
90	0.222	0.174	0.133	4.90 ± 0.19	4.76 ± 0.10	74.25 ± 1.32	16.86 ± 0.22	5.38 ± 0.10	3.08 ± 0.25	− 0.05	1.37
	0.256	0.199	0.152	6.69 ± 0.60	4.80 ± 0.29	73.86 ± 0.47	15.10 ± 0.30	5.36 ± 0.24	2.74 ± 0.28	− 0.04	2.04
	0.279	0.216	0.165	3.92 ± 0.38	4.62 ± 0.13	73.11 ± 0.56	15.01 ± 0.13	6.04 ± 0.21	2.64 ± 0.22	− 0.11	2.30
	0.321	0.246	0.190	5.83 ± 0.97	4.88 ± 0.29	70.40 ± 0.58	13.05 ± 0.27	6.52 ± 0.14	2.11 ± 0.08	− 0.03	2.71
110	0.148	0.119	0.090	4.34 ± 0.43	4.11 ± 0.37	74.01 ± 1.07	17.40 ± 0.33	6.15 ± 0.23	4.83 ± 1.02	− 0.06	0.47
	0.167	0.133	0.102	5.87 ± 0.80	5.62 ± 0.20	80.50 ± 1.53	18.63 ± 1.51	4.71 ± 0.39	2.53 ± 0.37	− 0.05	1.33
	0.173	0.135	0.102	5.32 ± 0.62	4.03 ± 0.21	74.72 ± 1.00	16.68 ± 0.34	7.26 ± 0.24	2.28 ± 0.15	− 0.08	0.82
	0.200	0.152	0.116	5.40 ± 0.98	3.91 ± 0.21	74.09 ± 0.26	15.50 ± 0.40	7.31 ± 026	1.61 ± 0.19	− 0.07	1.26
130	0.143	0.110	0.083	6.89 ± 0.67	3.13 ± 0.10	78.51 ± 0.54	19.54 ± 0.37	5.25 ± 0.15	3.17 ± 0.40	− 0.12	0.32

**Table 4:** The ratio of butyrate to acetate in three-stage continuous fermentations

*S*_f_	$D_{1}$	Butyrate/acetate		
(g/L)	(h^−1^)	First stage	Second stage	Third stage
90	0.222	1.38	2.43	3.13
	0.256	1.47	2.20	2.82
	0.279	1.14	1.88	2.49
	0.321	1.13	1.50	2.00
110	0.148	1.69	2.61	2.82
	0.167	2.37	3.20	3.96
	0.173	1.11	1.93	2.30
	0.200	1.07	1.75	2.12
130	0.143	3.20	3.86	3.72

In the third stage fermentation, remarkable 1,3-PDO concentrations ranging from 73.11 to 80.50 g/L were achieved with residual glycerol concentrations of 3.92–6.89 g/L. Meanwhile, the productivities of 1,3-PDO ranged from 0.32 to 2.71 g/(L·h), which are the lowest values in three-stage of continuous fermentation. At the same time, a decrease in biomass occurred in the third stage fermentation, resulting in negative biomass productivities (Table 3), indicating that the microorganism was undergoing a decline period. These results suggest that in the third stage fermentation, *C. butyricum* DL07 experienced the most severe inhibition, leading to the lowest productivity of 1,3-PDO (Table 3) and the highest ratio of butyrate to acetate (Table 4), when all product concentrations reached their peak levels.

In three-stage continuous fermentation, the overall 1,3-PDO productivity could reach 2.89–5.96 g/(L·h) while the yields distributed in 0.483–0.527 g/g (Table 5). The 1,3-PDO concentrations distributed in 73.11–80.50 g/L, which is comparable to some reported values in fed-batch fermentations [8, 11, 40], such as 80.5 g/L 1,3-PDO by two-pulsed continuous feeding in fed-batch fermentation [23]. Additionally, the main by-product was butyrate with concentrations ranging from 13.05 to 19.54 g/L. Acetate and lactate were also produced with the concentrations of approximately 4.71–7.31 g/L and 1.61–3.17 g/L, respectively. 80.05 g/L 1,3-PDO with an overall productivity of 3.67 g/(L·h) as the best concentration was achieved. Moreover, by-products including 18.63 g/L butyrate, 4.71 g/L acetate, and 2.53 g/L lactate were produced in this process. Notably, only 5.87 g/L glycerol was remained after fermentation, which would contribute to subsequent purification process of 1,3-PDO [41]. To our knowledge, the 1,3-PDO concentration of 80.05 g/L achieved in three-stage continuous fermentation is the highest reported value in continuous fermentation.

**Table 5:** The production of 1,3-PDO in three-stage continuous fermentations

*S*_f_	$D_{1}$	1,3-PDO (g/L)			*Y*_PDO_	*Q*_PDO_
(g/L)	(h^−1^)	First stage	Second stage	Third stage	(g/g)	(g/(L·h))
90	0.222	42.94 ± 0.71	65.16 ± 0.54	74.25 ± 1.32	0.516	4.40
	0.256	38.89 ± 0.70	61.54 ± 0.97	73.86 ± 0.47	0.527	4.99
	0.279	37.59 ± 0.95	60.28 ± 0.84	73.11 ± 0.56	0.514	5.36
	0.321	34.82 ± 1.06	57.79 ± 1.22	70.40 ± 0.58	0.515	5.96
110	0.148	50.85 ± 0.45	69.31 ± 0.83	74.01 ± 1.07	0.486	2.95
	0.167	48.37 ± 2.52	69.95 ± 2.57	80.50 ± 1.53	0.484	3.67
	0.173	46.30 ± 0.86	67.17 ± 0.57	74.72 ± 1.00	0.512	3.38
	0.200	41.42 ± 0.83	64.36 ± 0.90	74.09 ± 0.26	0.523	3.84
130	0.143	53.82 ± 0.71	75.13 ± 0.73	78.51 ± 0.54	0.512	2.89

### Kinetic models development for two-stage continuous 1,3-PDO production

The development of kinetic models for the first stage fermentation.

Kinetic models were proposed to characterize the continuous production process of 1,3-PDO. These models aimed to establish the kinetic relationship between major products (1,3-PDO and butyrate), substrate consumption, and cell growth during the first stage continuous fermentation. The mathematical Eqs. (1–3) represented the mass balance of products, substrate, and biomass in the continuous fermentation.

1 $$ \frac{{{\text{d}}X}}{{{\text{d}}t}} = \left( {\mu - D} \right)X, $$

2 $$ \frac{{{\text{d}}C_{{\text{s}}} }}{{{\text{d}}t}} = D\left( {S_{{\text{f}}} - C_{{\text{s}}} } \right) - q_{{\text{s}}} X, $$

3 $$ \frac{{{\text{d}}C_{{{\text{Pi}}}} }}{{{\text{d}}t}} = q_{{{\text{Pi}}}} X - DC_{{{\text{Pi}}}} \;\left( {{\text{Pi}} = 1,3{\text{-PDO}}, \;{\text{HBu}}} \right), $$

where *μ* is the specific growth rate of microbial cells (h^−1^), *X* is the biomass concentration (g/L), and *D* is the dilution rate (h^−1^). $C_{{\text{S}}}$, *S*_f_, *C*_P_ is the residual glycerol, initial glycerol, and product concentration (g/L), respectively. *q*_s_, *q*_P_ is the specific glycerol consumption rate and specific product formation rate (g/(g·h)), respectively.

Among these equations, the cell growth rate of *C. butyricum* DL07 is proposed based on the well-known Monod equation [42], a classical function mode used to describe the mathematical relationship between substrate concentration and microbial specific growth rate. Furthermore, the inhibition on cells by residual glycerol and main products concentration (1,3-PDO and butyrate) in the fermenter were also considered in Eq. (4), because both substrate and high product concentrations could inhibit microbial growth and metabolism. Product-inhibited cell growth is expressed in Eq. (4) using multiple product inhibition and growth modeling for *C. butyricum* [28, 43]. The linear relationships between *q*_s_ and *q*_P_ are presented by Eqs. (5)–(6), separately [44].

4 $$ \mu = \mu_{{\text{m}}} \frac{{C_{{\text{s}}} }}{{K_{{\text{S}}} + C_{{\text{S}}} }}\left( {1 - \frac{{C_{{\text{s}}} }}{{C_{{\text{s}}}^{*} }}} \right)\left( {1 - \frac{{C_{{{\text{PDO}}}} }}{{C_{{{\text{PDO}}}}^{*} }}} \right)^{2} \left( {1 - \frac{{C_{{{\text{HBu}}}} }}{{C_{{{\text{HBu}}}}^{*} }}} \right), $$

5 $$ q_{{\text{S}}} = m_{{\text{S}}} + \frac{\mu }{{Y_{X/S} }}, $$

6 $$ q_{{{\text{Pi}}}} = m_{{{\text{Pi}}}} + \mu \cdot Y_{{{\text{Pi}}/X}} \;\left( {{\text{Pi}} = {\text{PDO}},\;{\text{HBu}}} \right), $$

where *μ*_m_ is the maximum specific growth rate of cells (h^−1^), $C_{{\text{s}}}^{*}$ is the maximum residual substrate concentration (g/L), and *K*_s_ is the Monod saturation constant (g/L). $C_{{{\text{PDO}}}}$, $C_{{{\text{PDO}}}}^{*}$ is 1,3-PDO concentration in the bioreactor and the maximum 1,3-PDO concentration (g/L), respectively.$C_{{{\text{HBu}}}}$, $C_{{{\text{HBu}}}}^{*}$ is the butyrate and the maximum butyrate concentration (g/L), respectively. *m*_s_, *m*_p_ is the cell and product maintenance coefficient (g/(g·h)), respectively. *Y*_*X*/*S*_, *Y*_*P*/*X*_ is the biomass yield (g/g) and the coefficient of product per biomass (g/g), respectively.

In steady continuous fermentation of *K. peneumoniae*, the dilution rates are usually determined by the feeding rates of media without considering the addition of small amounts of alkaline solution used to neutralize acid production during fermentation [45, 46]. However, the dilution effect of the alkaline solution addition on the fermentation by *C. butyricum* should not be ignored, especially when high concentrations of 1,3-PDO are produced. Because significant amounts of butyric and acetate are produced, requiring a substantial quantity of alkaline solution to stabilize the pH [16, 20, 47]. Therefore, when conducting continuous fermentation with *C. butyricum* DL07, the dilution rates should take into account not only the supplement of the feeding media but also NaOH solution in kinetic systems. In a steady state of continuous fermentation, products, substrate, and cell concentrations remain unchanged over time. In this case, the rates of products formation, substrate consumption, and cell growth should be zero. As a result, left-hand sides of Eqs. (1)–(3) are equal to zero.

#### Estimation of parameters in the kinetic model

**Fig. 1:**
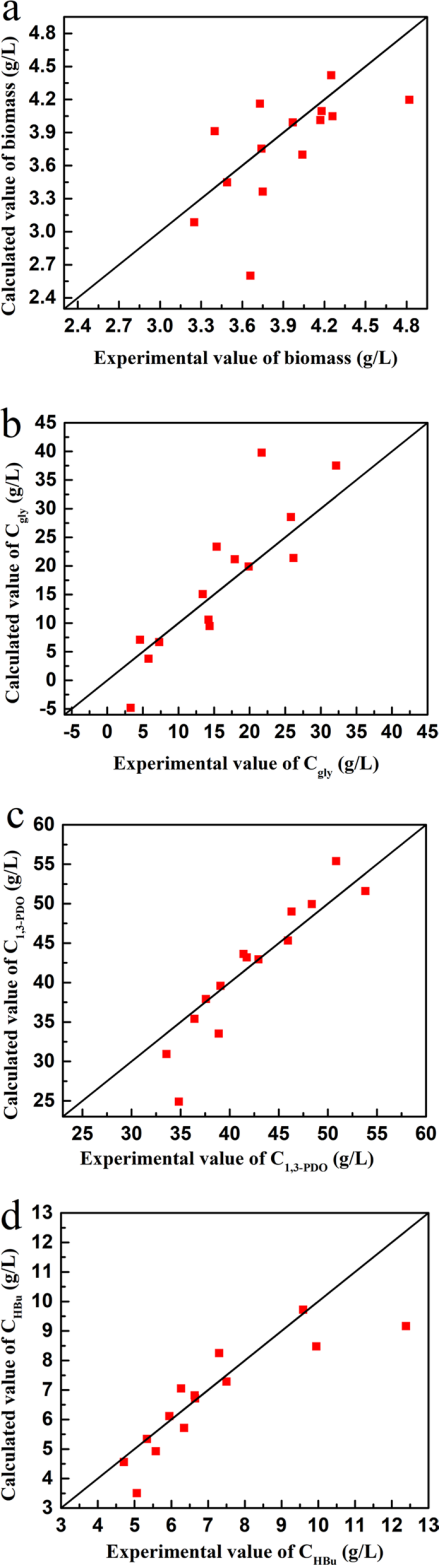
Comparison of biomass (**a**), glycerol (**b**), 1,3-PDO (**c**), butyrate (**d**) between experimental and predicted values at steady sates in the first stage continuous fermentations

The optimal parameters in the model were regressed with all the experimental data (14 groups) in the first stage continuous fermentation according to the relationship between Eqs. (1)–(6). The optimal parameters in the developed model and some reports are listed in Table 6. As shown in Table 6 and Fig. 1, for the biomass, residual glycerol, 1,3-PDO, and butyrate concentration, the mean relative errors were 7.8%, 43.8%, 6.2% and 9.5%, respectively. These results indicated that the microbial growth, substrate consumption and products formation could be well described by this developed kinetic model.

**Table 6:** The parameters in kinetic models of 1,3-PDO production

Parameters	Optimized value	References				
	This work^a^	[19]^a^	[52]^b^	[21]^a^	[37]^a^	[53]^c^
*μ*_max_ (h^−1^)	0.78	0.57	0.67	–	0.68	0.65
*K*_s_ (g/L)	0.116	1.66	0.026	–	–	12.8
$C_{{\text{S}}}^{*}$ (g/L)	183.6	–	187.8	–	181.7	181.7
$C_{{{\text{PDO}}}}^{*} \;{\text{(g/L)}}$	122.8	–	71.5	–	69.7	66.4
$C_{{{\text{Hbu}}}}^{*}$ (g/L)	27.54	–	–	–	24.7	–
$m_{{\text{s}}}$ (g/g/h)	1.99	–	0.20	0.91	–	0.23
$Y_{X/S}$ (g/g)	0.086	–	0.75	0.058	–	0.067
$m_{{{\text{PDO}}}}$ (g/g/h)	0.90	–	− 0.20	0.38	–	0.23
$Y_{{{\text{PDO}}/X}} \;{\text{(g/g)}}$	6.91	–	5.15	8.41	–	7.3
$m_{{{\text{HBu}}}}$ (g/g/h)	0.26	–	–	–	–	–
$Y_{{{\text{HBu}}/X}}$ (g/g)	0.58	–	–	–	–	–
MRE (%)	Biomass formation	7.8				
	Substrate consumption	43.8				
	1,3-PDO formation	6.2				
	Butyrate formation	9.5				

^a^*Clostridium butyricum*

^b^*Klebsiella pneumoniae*

^c^*Clostridium diolis*

**Fig. 2:**
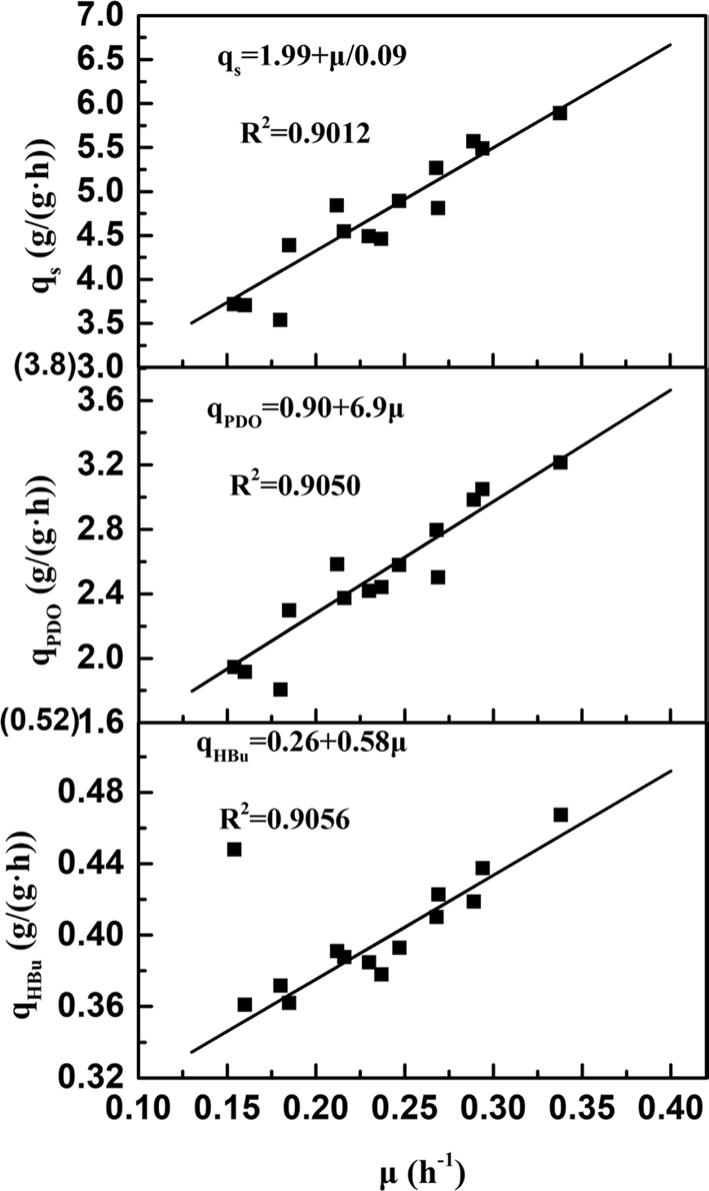
The relationship between specific glycerol consumption rate (*q*_s_), specific 1,3-PDO formation rate (*q*_PDO_), specific butyrate formation rate (*q*_HBu_) and the specific growth rates (*μ*) in the first stage continuous fermentation

Specifically, the model of substrate consumption (Fig. 2) exhibited an excellent linear correlation between the specific rates of glycerol consumption and microbial growth (*R*^2^ = 0.9012, *p* < 0.001). The cell maintenance coefficient (*m*_S_) and biomass yield (*Y*_*X*/*S*_) were 1.99 g/(g·h) and 0.09 g/g, separately. Meanwhile, good linear correlations were observed between the specific formation rates of products (1,3-PDO and butyrate) and the specific rates of cell growth (*R*^2^ = 0.9050, *p* < 0.001 for 1,3-PDO and *R*^2^ = 0.9056, *p* < 0.001 for butyrate) (Fig. 2). The yield coefficients of 1,3-PDO and butyrate formed by per biomass were 6.91 and 0.58 g/g, respectively, suggesting that these two products are growth-associated products [48, 49]. These results demonstrated that a stable and continuous 1,3-PDO production was achieved through *C. butyricum* DL07 fermentation if glycerol feeding concentrations (90–130 g/L) and feeding rates (0.143–0.321 h^−1^) are within the appropriate range. In the model of cell growth, the results indicated that the maximum specific growth of cells (*μ*_m_) was 0.78 h^−1^, which is slightly larger than the values in some reports (Table 6). In addition, high concentrations of 1,3-PDO, glycerol, and butyrate could inhibit microbial growth with the highest tolerance concentration of 122.8, 183.6 and 27.54 g/L, separately. Among these, 122.8 g/L 1,3-PDO as the maximum inhibitory concentration far exceed other reports values (71.5, 69.7, 66.4 g/L) in Table 6, but it is easy to understand since kinetic constants are related to both the species and culture conditions [29]. It had been reported that *C. butyricum* DL07 exhibited excellent capacity of 1,3-PDO production in fed-batch fermentation, especially its high-level concentration (104.8 g/L) and productivity (3.38 g/(L·h)) [20], which are much higher than those of other reports. Therefore, it is reasonable that the values of 1,3-PDO concentration ($C_{{{\text{PDO}}}}^{*}$) and the maximum specific growth of cells (*μ*_m_) are larger than those of reports. Other parameters were agreement with some reports (Table 6).

#### Mathematical relationships in the second stage continuous fermentation

**Fig. 3:**
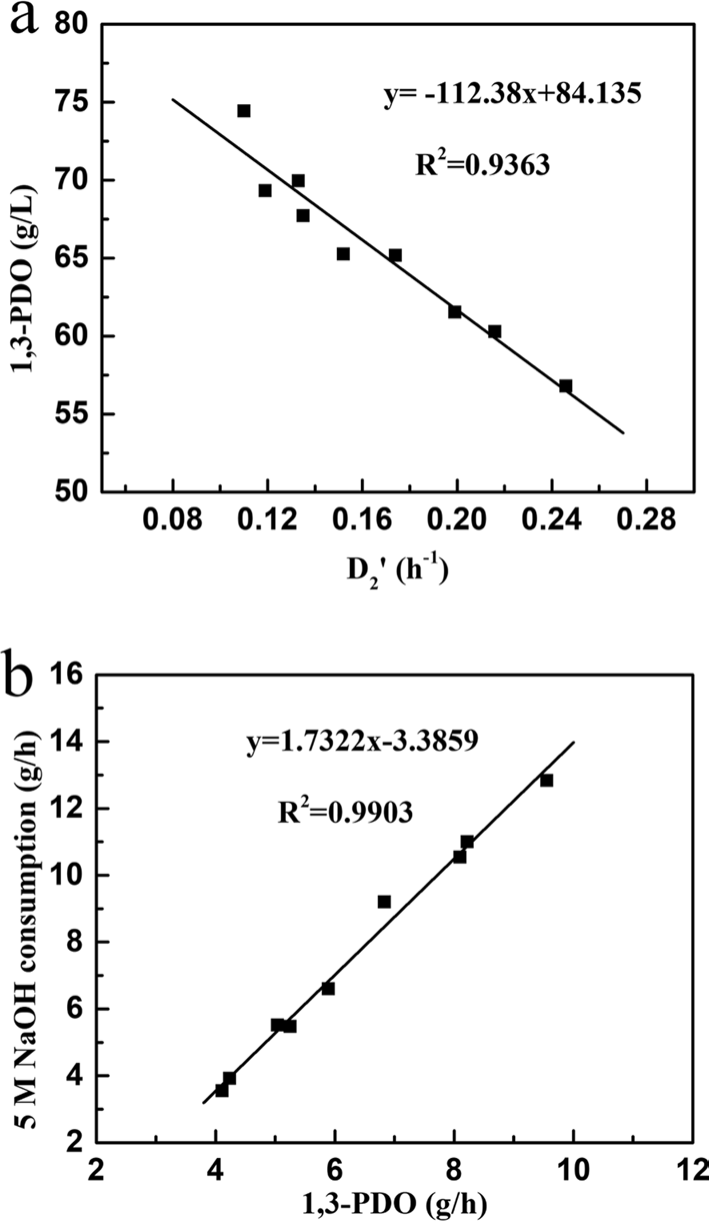
The relationship between 1,3-PDO concentration and dilution rate (**a**), the consumption rate of 5 M NaOH and the production rate of 1,3-PDO (**b**) in the second stage continuous fermentation

Two-stage continuous fermentation has many advantageous including reducing the use of control systems and human input compared to three-stage continuous fermentation. Therefore, it would be more potential for industrial applications if two-stage continuous fermentation could achieve high 1,3-PDO production (80 g/L). Some important mathematical relationships in the second stage fermentation were established to be used to predict the operation parameters for two-stage continuous fermentation. Fortunately, 1,3-PDO concentration was treated as a linear relationship to the dilution rate $\left( {D^{\prime}_{2} } \right)$ (*R*^2^ = 0.9362, *p* < 0.001) (Fig. 3a) in second stage continuous fermentation. Therefore, an undetermined dilution rate in the second stage could be predicted while a concentration of 1,3-PDO was set as the final target. Moreover, a good linear correlation between the increased amount of 1,3-PDO (g/h) and the addition of NaOH solution per hour (g/h) was also found (*R*^2^ = 0.9903, *p* < 0.001) (Fig. 3b), which can be applied in Eq. (8). The mass balance in the second stage fermentation was carried out in accordance with Eqs. (7)–(9) with some known parameters.

7 $$ D^{\prime}_{2} \times V_{2} = D^{\prime}_{1} \times V_{1} + F_{{2\;{\text{NaOH}}}} + F_{{2\;{\text{S}}}} , $$

8 $$ F_{{2\;{\text{NaOH}}}} = \left[ {1.7322 \times \left( {C_{{2\;{\text{PDO}}}} \times D^{\prime}_{2} \times V_{2} - C_{{1\;{\text{PDO}}}} \times D^{\prime}_{1} \times V_{1} } \right) - 3.3859} \right] \div \rho_{{{\text{NaOH}}}} , $$

9 $$ F_{{2\;{\text{Gly}}}} = \left[ {\left( {C_{{2\;{\text{PDO}}}} \times D^{\prime}_{2} \times V_{2} - C_{{1\;{\text{PDO}}}} \times D^{\prime}_{1} \times V_{1} } \right) \div Y_{{2\;{\text{PDO}}}} \div \omega_{{\text{S}}} + C_{{2\;{\text{S}}}} \times D^{\prime}_{2} \times V_{2} \div \omega_{{\text{S}}} - C_{{1\;{\text{S}}}} \times D^{\prime}_{1} \times V_{1} } \right] \div \rho_{{\text{S}}} , $$

where some parameters have been determined, such as $D^{\prime}_{2}$ = 0.037 h^−1^, $C_{{2\;{\text{S}}}}$ = 5 g/L, $\rho_{{ {\text{NaOH}}}} = 1.185 \times 10^{3} \;{\text{g}}/{\text{L}}; \;\rho_{{\text{S}}}$ = $1.129 \times 10^{3}$ g/L, $Y_{{2\;{\text{PDO}}}}$ = 0.48 g/g, $\omega_{{\text{S}}} { = 82}{\text{.5\% }}$. $V_{1}$, $V_{2}$ is the fermentation volume of the first and second stage fermentation (L), separately. $D^{\prime}_{1}$, $D^{\prime}_{2}$ is the dilution rate of the first and second stage fermentation (h^−1^), respectively. $F_{{2\;{\text{NaOH}}}}$, $F_{{2\;{\text{S}}}}$ is the consumption volume of 5 M NaOH and crude glycerol per hour in the second stage fermentation (L/h), respectively. $C_{{1\;{\text{PDO}}}}$, $C_{{2\;{\text{PDO}}}}$ is the 1,3-PDO concentration in the first and second stage fermentation (g/L), respectively. $C_{{2\;{\text{S}}}}$ is the glycerol concentration (g/L) and $Y_{{2\;{\text{PDO}}}}$ is the yield of 1,3-PDO (g/g) in the second stage fermentation. $\rho_{{{\text{NaOH}}}}$, $\rho_{{\text{S}}}$ is the density of 5 M NaOH and crude glycerol (g/L), respectively. $\omega_{{ {\text{Gly}}}}$ is the mass of pure glycerol in crude glycerol (%).

Some parameters are obtained by experimental data, such as $\rho_{{\text{S}}} , Y_{{2\;{\text{PDO}}}}$, and $\omega_{{\text{S}}}$. Meanwhile, other parameters are defined as target parameters. For example, a target concentration of 80 g/L for 1,3-PDO $\left( {C_{{2\;{\text{PDO}}}} } \right)$ is desired in the second stage fermentation. Moreover, the glycerol concentration after fermentation was expected to remain less than 5 g/L $\left( {C_{{2\;{\text{S}}}} } \right)$, because high glycerol concentrations can bring difficulties to the separation of 1,3-PDO. By considering these objectives, the volume ratio of the two fermenters ($V_{2}$/$V_{1}$) could be determined as the only parameter according to Eqs. (7)–(9).

### Kinetics-based development of two-stage continuous fermentation

#### Optimization of 1,3-PDO production in the first stage fermentation

This developed kinetic model, designed to describe the production of 1,3-PDO, can serve as a valuable tool to optimize the operation conditions, such as glycerol feed concentration and feeding rate of media [50, 51]. In the first stage fermentation, the productivities of biomass and 1,3-PDO are treated as the most important factors [52], as they directly determine the level of 1,3-PDO concentration in the subsequent second stage fermentation. Fortunately, the microbial growth is positively correlated with 1,3-PDO production in *C. butyricum* DL07. Consequently, the aim of first stage fermentation is to achieve the highest 1,3-PDO productivity (*Q*_PDO_) and biomass productivity (*Q*_Biom_).

The optimization process was performed, employing Eq. (10), to determine the glycerol feeding concentration and feeding rates of media to maximize the productivities of 1,3-PDO and biomass.

$$ {\text{Max}}:Q_{{{\text{PDO}}}} = (DC_{{{\text{PDO}}}} ) $$

10 $$ \left\{ \begin{gathered} \left[ {\mu_{{\text{m}}} \frac{{C_{{\text{s}}} }}{{K_{{\text{S}}} + C_{{\text{S}}} }}\left( {1 - \frac{{C_{{\text{s}}} }}{{C_{{\text{s}}}^{*} }}} \right)\left( {1 - \frac{{C_{{{\text{PDO}}}} }}{{C_{{{\text{PDO}}}}^{*} }}} \right)^{2} \left( {1 - \frac{{C_{{{\text{HBu}}}} }}{{C_{{{\text{HBu}}}}^{*} }}} \right) - D} \right]X = 0 \hfill \\ D\left( {S_{{\text{f}}} - C_{{\text{S}}} } \right) - \left( {m_{{\text{S}}} + \frac{\mu }{{Y_{X/S} }}} \right)X = 0 \hfill \\ \left( {m_{{{\text{PDO}}}} + \mu \cdot Y_{{{\text{PDO}}/X}} } \right)X - DC_{{{\text{PDO}}}} = 0 \hfill \\ \left( {m_{{{\text{HBu}}}} + \mu \cdot Y_{{{\text{HBu}}/X}} } \right)X - DC_{{{\text{HBu}}}} = 0 \hfill \\ S_{{\text{f}}} \in \left( {60, \, 184} \right) \hfill \\ D \in \left( {0.1, \, 1.0} \right),\;{\text{span}}\;0.01 \hfill \\ \end{gathered} \right.. $$

**Fig. 4:**
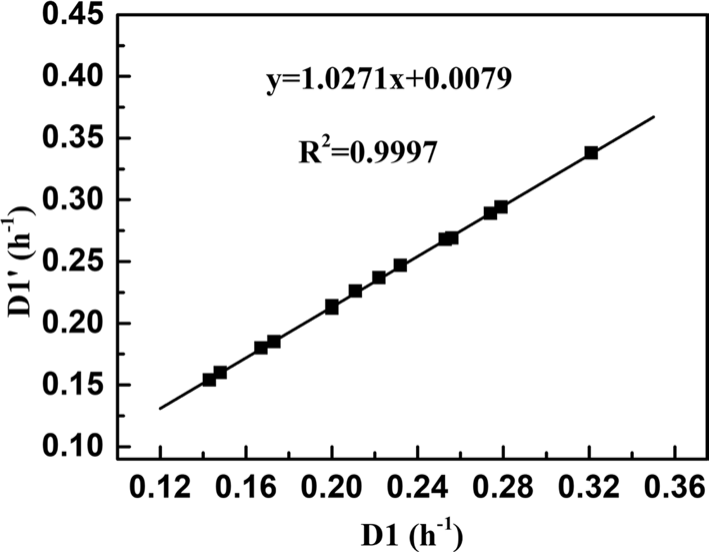
The mathematical relation between the overall dilution rates $\left( {D^{\prime}_{1} } \right)$ and the feeding rates of media $\left( {D_{1} } \right)$ in the first stage continuous fermentation

As shown in Table 7, when there are no constraints on the concentrations of remaining glycerol and 1,3-PDO, the highest predicted 1,3-PDO productivity would reach up to 11.26 g/(L·h) when the feeding glycerol concentration and feeding dilution rate were 92 g/L and 0.341 h^−1^, respectively. Meanwhile, the highest biomass productivity was predicted to reach 1.21 g/(L·h), accompanied by 31.45 g/L 1,3-PDO and 4.5 g/L butyrate. Although the predicted maximum 1,3-PDO productivity (11.26 g/(L·h)) is slightly lower than the experimental value (11.77 g/(L·h)) (Table 1), these two values and the fermentation conditions are very similar. Therefore, this small error is considered acceptable. To verify the accuracy of the model, two additional fermentation conditions were predicted based on the random restriction of glycerol and 1,3-PDO concentrations in the fermenter (8 ≤ $C_{{\text{S}}}$ ≤ 20 g/L and *C*_PDO_ ≥ 45 g/L; 8 ≤ $C_{{\text{S}}}$ ≤ 20 g/L and *C*_PDO_ ≥ 55 g/L) for the highest 1,3-PDO productivity. When a target 1,3-PDO concentration was set to exceed 45 g/L, the highest predicted productivities of 1,3-PDO and biomass were 9.73 and 0.88 g/(L·h), respectively. Accordingly, the fermentation conditions are predicted to be 102 g/L feeding glycerol with a feeding dilution rate of 0.200 h^−1^ (Table 7). If more than 55 g/L 1,3-PDO was desired, the predicted highest productivities of 1,3-PDO and biomass are 7.71 and 0.60 g/(L·h), separately, while the feeding glycerol concentration reaches 126 g/L with a low feeding dilution rate of 0.131 h^−1^. The specific concentrations of butyrate and glycerol are listed in the Table 7. During the experimental manipulation, the medium feeding rates, rather than the dilution rates in the continuous fermentation, can be manually set. Therefore, the predicted optimal dilution rates need to be converted to the feeding rates of media considering the actual experimental manipulation. Fortunately, a good linear correlation was observed between dilution rates and feeding rates of media in first stage fermentation (*R*^2^ = 0.9997, *p* < 0.001) (Fig. 4). Consequently, the feeding rates of media are listed in Table 7.

**Table 7:** Kinetics-based design of two-stage continuous fermentations and their experimental verification

Conditions	*C*_f_ (g/L)	$D_{1}$	$D^{\prime}_{1}$	Groups	$C_{S}$	Biomass	1,3-PDO	Butyrate	*Q*_PDO_	*Q*_Biomass_	MRE (%)	$V_{2}$/$V_{1}$
		(h^−1^)			(g/L)				(g/(L·h))			
No constraint	92	0.341	0.358	PV	30.12	3.38	31.45	4.50	11.26	1.12	4.2	11.9
				EV	28.28	3.60	31.72	4.93	11.36	1.29		–
${8 } \le C_{{\text{S}}}$ ≤ 20 g/L $C_{{{\text{PDO}}}}$ ≥ 45 g/L	102	0.200	0.214	PV	10.11	4.09	45.49	7.34	9.73	0.88	12.5	6.9
				EV	7.04	3.95	44.62	7.42	9.55	0.85		–
${8 } \le C_{{\text{S}}}$ ≤ 20 g/L $C_{{{\text{PDO}}}}$ ≥ 55 g/L	126	0.131	0.142	PV	9.21	4.16	55.06	10.00	7.82	0.59	10.7	4.4
				EV	7.64	4.24	54.27	12.34	7.71	0.60		–

PV: the predicted values; EV: the experimental values; *V*_2_/*V*_1_: the predicted volume ratio of two reactors in a two-stage fermentation to achieve 80 g/L 1,3-PDO at the second stage

#### Optimization of two-stage continuous fermentation

The optimal fermentation conditions, as predicted by the developed model, were implemented in the first stage fermentation of two-stage continuous fermentation. Moreover, employing the mathematical relationships established for the second stage fermentation, some operational parameters were predicted for a high 1,3-PDO concentration in two-stage continuous fermentation. For example, the dilution ratio of the two bioreactors ($D^{\prime}_{2}$/$D^{\prime}_{1}$) and the volume ratio of the two fermenters ($V_{2}$/$V_{1}$) would play important parts in controlling the retention time of microorganisms during the fermentation, ultimately determining the 1,3-PDO production in two-stage fermentation [52].

If a target of 80 g/L 1,3-PDO was required in two-stage continuous fermentation, the appropriate dilution rate in the second stage fermentation was determined to be 0.037 h^−1^ according to the relationships (Fig. 3a). Subsequently, the volume ratio of the two fermenters ($V_{2}$/$V_{1}$) would be calculated by the Eqs. (7–9). Of course, the fermentation volume of the second fermenter would depend on the specific conditions of first stage fermentation. Therefore, based on the predicted conditions in the first stage fermentation, the corresponding volume ratios of two fermenters ($V_{2}$/$V_{1}$) in two-stage continuous fermentation were determined and listed in Table 7 for producing a target 1,3-PDO of 80 g/L. If there is in absence of restrictions, the volume ratio of two fermenters ($V_{2}$/$V_{1}$) of 11.9 could achieve 80 g/L 1,3-PDO by two-stage continuous fermentation accompanied by the highest 1,3-PDO productivity (11.26 g/(L·h)) in the first stage fermentation. If the desired 1,3-PDO concentrations in the first stage fermentation were 45 and 55 g/L, the volume ratios of the two fermenters ($V_{2}$/$V_{1}$) would be 6.9 and 4.4, respectively. The different ratios of the two fermenters indicate that the same target 1,3-PDO concentration could be obtained in the second stage fermentation under different fermentation conditions ($V_{1}$, *S*_f_, and $D_{1}$) in the first stage fermentation. These values were predicted theoretically, so it should be experimentally verified. After that, once the yield of 1,3-PDO was required by a company, the relevant volume of two fermenters, along with others parameters, could be directly designed using these equations.

#### Experimental verification of the kinetics-based two-stage continuous fermentations

For the first stage fermentation, experiments were performed separately under these three optimized fermentation conditions described in Table 7. Remarkably, the actual fermentation results closely matched the predicted values for 1,3-PDO, biomass, butyrate, and glycerol concentrations under these three predicted experimental conditions. The mean relative error between the experimental and predicted values are 4.2%, 12.5%, and 10.7%, respectively. These results demonstrate the suitability of the developed mode for optimizing fermentation conditions and predicting the products formation. In the experiment, the highest productivities of 1,3-PDO and biomass were 11.36 and 1.29 g/(L·h), respectively. Comparatively, the maximum productivities of 1,3-PDO and biomass in previous experiments were 11.77 and 1.24 g/(L·h), respectively (Table 1). This suggests that the fermentation condition used in previous experiment, can also be considered an optimal fermentation condition. It is understandable that several fermentation conditions could achieve almost the highest productivity. Therefore, an initial glycerol concentration of around 90 g/L and a feeding dilution rate approximately 0.32–0.34 h^−1^, are deemed suitable for the first stage continuous fermentation as the optimal condition.

**Fig. 5:**
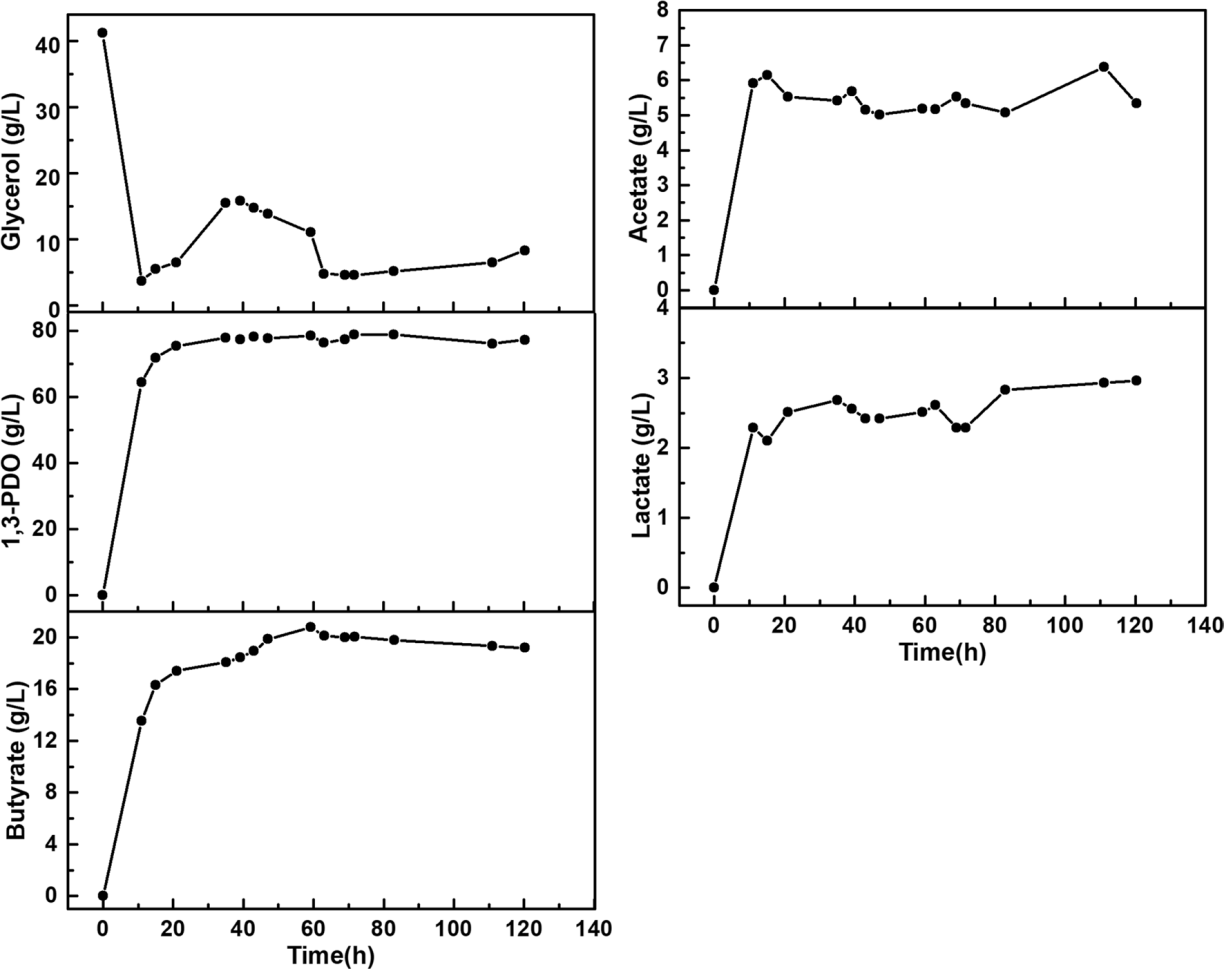
The experimental results of a two-stage continuous fermentation under the model-predicted conditions (*S*_f_ = 126 g/L, $D_{1}$ = 0.13 h^−1^, $V_{2}$/$V_{1}$ = 4.4)

According to the successful experimental verification in the first sage fermentation, the second stage fermentation was also performed to validate the feasibility of two-stage fermentation process. Based on a fermentation volume of 1 L in the first stage fermentation, the volume of the second fermenter was determined to be 11.9, 6.9 or 4.4 L (Table 7). Considering the available working volumes of the fermenters in our lab, a fermentation volume of 4.4 L was determined to perform the experiment. Therefore, the first stage fermentation was initiated under a feeding glycerol concentration of 126 g/L and a feeding dilution rate of 0.131 h^−1^. Then the second stage fermentation proceed with the fermentation with a broth volume of 4.4 L and a dilution rate of 0.037 h^−1^. The experimental results (Fig. 5) indicated that the average concentration of 1,3-PDO reached 77.46 g/L with 19.33 g/L butyrate, 5.40 g/L acetate and 2.58 g/L lactate. Apparently, the experimental concentration of 1,3-PDO was close to the target concentration of 80 g/L, with a small error (3.2%) compared to the predicted value. However, the cell productivity in the first stage fermentation was only 0.60 g/(L·h) (Table 7) suggesting that the high product concentrations (54.27 g/L 1,3-PDO and 12.34 g/L butyrate) in the first stage fermentation inhibited microbial growth and metabolism. In Fig. 5, the residual glycerol concentration exhibited fluctuations because the feeding rates of glycerol had been constantly artificially adjusted during fermentation. Finally, the residual glycerol could be maintained about 5 g/L during the steady state (after 63 h), which is conductive to the purification of 1,3-PDO. It is believed that it would be possible to produce more than 80 g/L 1,3-PDO in two-stage continuous fermentation, if the cells are in a robust growth state in the first stage fermentation. Therefore, a feeding glycerol of 92 g/L and a feeding rate of 0.341 h^−1^ in the first stage fermentation are considered as the optimal fermentation condition for achieving high concentrations of 1,3-PDO (≥ 80 g/L) in two-stage continuous fermentation. In addition, the predicted volume ratio of the two fermenters ($V_{2}$/$V_{1}$) would be 11.9. In the future, the automatic implementation of two-stage continuous fermentation with the developed kinetic model would hold great potential for the industrial-scale production of 1,3-PDO.

## Conclusions

A three-stage continuous fermentation process was successfully performed to produce high concentration of 1,3-PDO by *C. butyricum* DL07. In the first stage fermentation, the strain exhibited excellent performance in terms of 1,3-PDO productivity (8.14–11.77 g/(L·h)) and cell growth (0.90–1.24 g/(L·h)) when glycerol concentrations in feeding medium ranged from 90 to 100 g/L, with feeding rates of 0.20–0.32 h^−1^. Given the robust cell growth in the first stage fermentation, the second stage fermentation was initiated to enhance 1,3-PDO production. As a result, the 1,3-PDO concentration was increased by 18.46–22.97 g/L in the second stage fermentation using the matching glycerol feeding rates while maintaining a glycerol concentration of approximately 20 g/L. Subsequently, the third stage fermentation was proceeded for the highest 1,3-PDO concentration and the lowest residual glycerol concentration (< 7 g/L). Finally, the highest 1,3-PDO concentration reached 80.05 g/L at a remaining glycerol concentration of 5.87 g/L. The overall productivity and yield of 1,3-PDO reached up to 3.67 g/(L·h) and 0.48 g/g, respectively. Additionally, by-products such as butyrate (18.63 g/L), acetate (4.71 g/L), and lactate (2.53 g/L) were produced during this process. To achieve the optimization of 1,3-PDO production in two-stage fermentation, a new kinetic model was developed based on the concentrations of biomass, substrate, 1,3-PDO, and butyrate. The optimal fermentation condition for the first stage fermentation (glycerol feeding concentration of 92 g/L and feeding rate of 0.341 h^−1^) was predicted to obtain the highest 1,3-PDO productivity based on the kinetic model. Furthermore, under the above fermentation condition, the volume ratio of the two fermenters ($V_{2}$/$V_{1}$ ≥ 11.9) was predicted to produce more than 80 g/L 1,3-PDO by two-stage continuous fermentation. Finally, the reliability of the developed model was confirmed through experiments by one- and two-stage continuous fermentations. This optimized two-stage continuous fermentation, coupled with the developed kinetic model by *C. butyricum* DL07, are valuable for the industrial continuous production of 1,3-PDO. It also provides a reference for the production of other bio-chemicals by continuous fermentation.

## Methods

### Materials

The crude glycerol used in this research includes 82% glycerol, 12% moisture, 4.3% ash and was purchased from Dongma Oil and Fats (Zhangjiagang Free Trade Zone) Co. Ltd., China. Its pH value was 6.55. It was produced through the hydrolysis of palm oil. The yeast extract (FM888) includes 11% ash, 10% total nitrogen, and 2% NaCl and was purchased from Angel Yeast Co. Ltd., Yichang, China.

### Microorganism and culture media

In this study, *C. butyricum* DL07 was employed and preserved at − 70 °C in our lab. The selection and preservation methods of this strain were referred to our previous work [20]. The seed culture and initial fermentation medium will also follow our previous report [20]. In the first-stage fermentation, the feeding medium was similar with the initial fermentation medium except that different glycerol concentrations. The crude glycerol was fed in the second and third stage fermentation if necessary.

### Seed culture and a three-stage continuous fermentation

Anaerobic seed cultures were conducted in the anaerobic bottle (250 mL). 100 mL of seed medium was prepared with nitrogen aeration and sterilized at 121 °C for 15 min. The stored strain in refrigerator was firstly activated in a bottle at an inoculation ratio of 4% (v/v), shaking at 200 rpm, 37 °C for 12 h. Subsequently, 2% of activated seed was again inoculated into seed medium under the same cultivation conditions to serve as inoculum for subsequent fermentation.

A three-stage continuous fermentation was implemented in three 5.0 L bioreactors (Baoxing Biotech, Shanghai, China) containing the appropriate volume of initial fermentation medium. Both the initial fermentation medium in bioreactor and feeding medium were sterilized at 121 °C for 15 min. Before inoculating, to achieve the anaerobic environment, the initial fermentation medium was aerated with nitrogen at 0.1 vvm for 1 h. Thereafter, nitrogen supply was adjusted to 0.05 vvm for the whole fermentation process. In the first stage continuous fermentation, 100 mL of seed broths [inoculation rate of 10% (v/v)] were transferred into the first bioreactor containing 1 L fermentation medium. Once the initial glycerol concentration in the bioreactor was consumed to 20 g/L, fresh feeding medium was pumped into the first bioreactor at a set rate. Simultaneously, fermentation broth was removed from bioreactor at a certain rate to the second bioreactor so that 1 L of fermentation broths was always maintained in the bioreactor. Similarly, the fermentation broth of 1.5 L in the second bioreactor was maintained while pumping out the excessive broth at a constant rate, and the third bioreactor contained 2 L fermentation broth. The final fermentation broth was obtained from the third stage fermentation. Crude glycerol was directly fed at different rates to pursue approximately 20 g/L in the second stage fermentation. The feeding rate of crude glycerol depended on cell metabolism in the third bioreactor so as to keep a low glycerol concentration (< 7 g/L) in the final fermentation broth. The three-stage continuous fermentation ran automatically at 37 °C and a pH of 7.0. At the same time, 5 mol/L NaOH solution was used to control a predetermined pH. The stirring rate was set at 250 rpm. A steady state in each stage of fermentation was achieved with 7 times volume exchanges of fermentation broth in each bioreactor. The feeding rates of media $\left( {D_{1} } \right)$ and dilution rates of each fermenter ($D^{\prime}_{1}$, $D^{\prime}_{2}$, $D^{\prime}_{3}$) were mean values of three times measure by a 250 mL cylinder.

The kinetics-based two-stage continuous fermentations were experimentally verified according to the above three-stage continuous fermentation without the third bioreactor. The feeding rate of medium and volume of fermentation broth were determined by the optimization of fermentation kinetics based on the three-stage continuous fermentations.

### Analytical methods

The glycerol content of crude glycerol was analyzed using HPLC. Ash determination of crude glycerol was performed at 800 °C in a high temperature furnace.

During the fermentation process, 6 mL fermentation broth was periodically sampled from the bioreactors and used to detect biomass, residual glycerol, and metabolites concentration.

The biomass was expressed in cell dry weight (CDW) per liter. It was calculated by the optical density (OD) of fermentation broth. The OD of samples was measured use a UV–visible spectroscopy at 650 nm. After then cell sediments were collected and washed twice with saline solution by centrifugation of sample at 12,000 rpm for 20 min. Finally, the cell sediments were dried at 65 °C overnight to a constant weight. The linear equations between biomass (g/L) and OD value were shown as follows:

11 $$ C_{{1\;{\text{Biom}}}} = {\text{OD}}_{1,650} \times 0.294, $$

12 $$ C_{{2\;{\text{Biom}}}} = {\text{OD}}_{2,650} \times 0.366, $$

13 $$ C_{{3\;{\text{Biom}}}} = {\text{OD}}_{3,650} \times 0.368, $$

where OD_*n*,650_ and *C*_*n* Biom_ (*n* = 1, 2, 3) represent OD at 650 nm and CDW (g/L) of fermentation broth in the three-stage fermentation, respectively.

The residual glycerol concentration can also be tested quickly according to a report [30]. The specific metabolites concentrations including 1,3-PDO, butyrate, acetate, and lactate were analyzed via high performance liquid chromatography (HPLC). Before HPLC analysis, the clarified fermentation broths of samples were obtained by centrifugation at 12,000 rpm for 10 min. After then chloroform mixed with the clarified fermentation broth at 1:1 and centrifugated to remove the proteins. Finally, the above supernatant was filtered through 0.22-μm membrane filter after appropriate dilution and analyzed for metabolites. The HPLC systems include an auto-sampler delivery system (Waters 2707), a pump (Waters 1515), a differential refractometer detector (Waters 2414), and an Aminex HPX 87H column (300 mm × 7.8 mm; Bio-Rad). The detector temperatures, column temperature and detection volume were set at 35 °C, 65 °C and 20 μL, respectively. 5 mmol/L H_2_SO_4_ was served as tested mobile phase at a flow of 0.6 mL/min. The yield and productivity of 1,3-PDO, and biomass were analyzed using the following equations.

14 $$ Q_{{1\;{\text{PDO}} }} = C_{{1\;{\text{PDO}}}} \times D^{\prime}_{1} , $$

15 $$ Q_{{1\;{\text{Biom}} }} = C_{{1\;{\text{Biom}}}} \times D^{\prime}_{1} , $$

16 $$ Q_{{2\;{\text{PDO}} }} = \left( {C_{{2\;{\text{PDO}}}} - \frac{{C_{{1\;{\text{PDO}}}} \times F^{\prime}_{1} }}{{F^{\prime}_{2} }}} \right) \times D^{\prime}_{2} , $$

17 $$ Q_{{2\;{\text{Biom}}}} = \left( {C_{{2\;{\text{Biom}}}} - \frac{{C_{{1\;{\text{Biom}}}} \times F^{\prime}_{1} }}{{F^{\prime}_{2} }}} \right) \times D^{\prime}_{2} , $$

18 $$ Q_{{3\;{\text{PDO}} }} = \left( {C_{{3\;{\text{PDO}}}} - \frac{{C_{{2\;{\text{PDO}}}} \times F^{\prime}_{2} }}{{F^{\prime}_{3} }}} \right) \times D^{\prime}_{3} , $$

19 $$ Q_{{3\;{\text{Biom}} }} = \left( {C_{{3\;{\text{Biom}}}} - \frac{{C_{{2\;{\text{Biom}}}} \times F^{\prime}_{2} }}{{F^{\prime}_{3} }}} \right) \times D^{\prime}_{3} , $$

20 $$ Y_{{{\text{PDO}}}} = \frac{{C_{{3\;{\text{PDO}}}} \times F^{\prime}_{3} }}{{S_{{{\text{f}}\;{\text{gly}} }} \times F_{1 } + m_{2 } + m_{3 } - C_{{3\;{\text{gly}} }} \times F^{\prime}_{3} }}, $$

21 $$ Q_{{ {\text{PDO}}}} = C_{{3\;{\text{PDO}}}} \times \frac{{F^{\prime}_{3} }}{{V_{1 } + V_{2 } + V_{3 } }}, $$

where $C_{{n\;{\text{Biom}}}}$, $C_{{n\;{\text{gly}}}}$, and $C_{{n\;{\text{PDO}}}}$ (*n* = 1, 2, 3) are the biomass, glycerol, and 1,3-PDO concentrations at each stage fermentation (g/L), respectively. $Q_{{n\;{\text{Biom}}}}$ and $Q_{{n\;{\text{PDO}}}}$ (*n* = 1, 2, 3) are the productivities of biomass and 1,3-PDO at each stage fermentation (g/(L·h)), respectively. $Q_{{ {\text{PDO}}}}$ is the overall productivity of 1,3-PDO (g/(L·h)) and $Y_{{ {\text{PDO}}}}$ is the overall yield of 1,3-PDO (g/g) in three-stage continuous fermentation. $m_{2 }$ and $m_{3 }$ are the feeding amount of glycerol per hour at the second and third stage fermentation (g/h), respectively. $V_{n }$ (*n* = 1, 2, 3) is the volume of fermentation broth at each stage fermentation. $F^{\prime}_{n}$ (*n* = 1, 2, 3) is fermentation broth outflow rate from each stage fermentation.

### Kinetic model and parameters estimation

A general method of Runge–Kutta (4-order) was used to solve the developed model. The *fmincon* in MATLAB (R2018b) was used for the optimization of parameters and fermentation conditions. The developed model was evaluated by the mean relative error (MRE) between the experimental and predicted values.

## Data Availability

All data generated and/or analyzed during this study are available from the corresponding author upon reasonable request.

## References

[1] Anitha M, Kamarudin SK, Kofli NT (2016). The potential of glycerol as a value-added commodity. Chem Eng J.

[2] Barbirato F, Himmi EH, Conte T, Bories A (1998). 1,3-Propanediol production by fermentation: an interesting way to valorize glycerin from the ester and ethanol industries. Ind Crop Prod.

[3] Chilakamarry CR, Sakinah AMM, Zularisam AW, Pandey A (2021). Glycerol waste to value added products and its potential applications. Syst Microbiol Biomanuf.

[4] Fokum E, Zabed HM, Yun J, Zhang G, Qi X (2021). Recent technological and strategical developments in the biomanufacturing of 1,3-propanediol from glycerol. Int J Environ Sci Technol.

[5] Ruan M, Luan H, Wan G, Shen M (2019). Bio-polyols synthesized from bio-based 1,3-propanediol and applications on polyurethane reactive hot melt adhesives. Ind Crop Prod.

[6] Martins FF, Liberato VDSS, Ribeiro CMS, Coelho MAZ, Ferreira TF (2020). Low-cost medium for 1,3-propanediol production from crude glycerol by *Clostridium butyricum*. Biofuels Bioprod Biorefin.

[7] Kluge M, Pérocheau Arnaud S, Robert T (2019). 1,3-Propanediol and its application in bio-based polyesters for resin applications. Chem Africa.

[8] Lan Y, Feng J, Guo X, Fu H, Wang J (2021). Isolation and characterization of a newly identified *Clostridium butyricum* strain SCUT343-4 for 1,3-propanediol production. Bioprocess Biosyst Eng.

[9] Sun Y, Shen J, Yan L, Zhou J, Jiang L, Chen Y, Yuan J, Feng E, Xiu Z (2018). Advances in bioconversion of glycerol to 1,3-propanediol: prospects and challenges. Process Biochem.

[10] Zhu Y, Wang Y, Gao H, Wang H, Wan Z, Jiang Y, Xin F, Zhang W, Jiang M (2021). Current advances in microbial production of 1,3-propanediol. Biofuel Bioprod Biorefin.

[11] Maina S, Kachrimanidou V, Ladakis D, Papanikolaou S, Castro AM, Koutinas A (2019). Evaluation of 1,3-propanediol production by two *Citrobacter freundii* strains using crude glycerol and soybean cake hydrolysate. Environ Sci Pollut Res.

[12] Ju J, Wang D, Heo S, Kim M, Seo J, Kim Y, Kim D, Kang S, Kim C, Oh B (2020). Enhancement of 1,3-propanediol production from industrial by-product by *Lactobacillus reuteri* CH53. Microb Cell Fact.

[13] Shen J, Zhou J, Fu H, Mu Y, Sun Y, Xu Y, Xiu Z (2016). A *Klebsiella pneumoniae* bacteriophage and its effect on 1,3-propanediol fermentation. Process Biochem.

[14] Zhou J, Shen J, Wang X, Sun Y, Xiu Z (2020). Metabolism, morphology and transcriptome analysis of oscillatory behavior of *Clostridium butyricum* during long-term continuous fermentation for 1,3-propanediol production. Biotechnol Biofuels.

[15] Wang X, Zhou J, Sun Y, Xiu Z (2019). Bioconversion of raw glycerol from waste cooking-oil-based biodiesel production to 1,3-propanediol and lactate by a microbial consortium. Front Bioeng Biotechnol.

[16] Tee ZK, Jahim JM, Tan JP, Kim BH (2017). Preeminent productivity of 1,3-propanediol by *Clostridium butyricum* JKT37 and the role of using calcium carbonate as pH neutraliser in glycerol fermentation. Bioresour Technol.

[17] Yang X, Kim DS, Choi HS, Kim CK, Thapa LP, Park C, Kim SW (2017). Repeated batch production of 1,3-propanediol from biodiesel derived waste glycerol by *Klebsiella pneumoniae*. Chem Eng J.

[18] Chatzifragkou A, Papanikolaou S, Kopsahelis N, Kachrimanidou V, Dorado M, Koutinas A (2014). Biorefinery development through utilization of biodiesel industry by-products as sole fermentation feedstock for 1,3-propanediol production. Bioresour Technol.

[19] Zhang A, Huang S, Zhuang X, Wang K, Yao C, Fang B (2019). A novel kinetic model to describe 1,3-propanediol production fermentation by *Clostridium butyricum*. AIChE J.

[20] Wang X, Zhou J, Shen J, Zheng Y, Sun Y, Xiu Z (2020). Sequential fed-batch fermentation of 1,3-propanediol from glycerol by *Clostridium butyricum* DL07. Appl Microbiol Biotechnol.

[21] Zhou J, Shen J, Wang X, Sun Y, Xiu Z (2018). Stability and oscillatory behavior of microbial consortium in continuous conversion of crude glycerol to 1,3-propanediol. Appl Microbiol Biot.

[22] Papanikolaou S, Ruiz-Sanchez P, Pariset B, Blanchard F, Fick M (2000). High production of 1,3-propanediol from industrial glycerol by a newly isolated *Clostridium butyricum* strain. J Biotechnol.

[23] Wong N, Jantama K, Cassan C, Taillandier P (2023). Process optimization of metabolically engineered *Escherichia coli* NSK015 fermentation for progressive improvement of 1,3-propanediol production. J Chem Technol Biotechnol.

[24] Wong N, Jantama K (2022). Engineering *Escherichia coli* for a high yield of 1,3-propanediol near the theoretical maximum through chromosomal integration and gene deletion. Appl Microbiol Biotechnol.

[25] Petrache HI, Tristram-Nagle S, Harries D, Kučerka N, Nagle JF, Parsegian VA (2006). Swelling of phospholipids by monovalent salt. J Lipid Res.

[26] Zhou J, Shen J, Jiang L, Sun Y, Mu Y, Xiu Z (2017). Selection and characterization of an anaerobic microbial consortium with high adaptation to crude glycerol for 1,3-propanediol production. Appl Microbiol Biotechnol.

[27] Colin T, Bories A, Lavigne C, Moulin G (2001). Effects of acetate and butyrate during glycerol fermentation by *Clostridium butyricum*. Curr Microbiol.

[28] Zeng AP, Ross A, Biebl H, Tag C, Günzel B, Deckwer WD (1994). Multiple product inhibition and growth modeling of *Clostridium butyricum* and *Klebsiella pneumoniae* in glycerol fermentation. Biotechnol Bioeng.

[29] Zhu C, Fang B, Wang S (2016). Effects of culture conditions on the kinetic behavior of 1,3-propanediol fermentation by *Clostridium butyricum* with a kinetic model. Bioresour Technol.

[30] Wang JF, Xiu ZL, Fan SD (2001). Determination of glycerin concentration during the fermentation of glycerin to 1, 3-propanediol. Ind Microbiol.

[31] Reimann A, Biebl H, Deckwer WD (1998). Production of 1,3-propanediol by *Clostridium butyricum* in continuous culture with cell recycling. Appl Microbiol Biotechnol.

[32] González-Pajuelo M, Andrade JC, Vasconcelos I (2005). Production of 1,3-propanediol by *Clostridium butyricum* VPI 3266 in continuous cultures with high yield and productivity. J Ind Microbiol Biotechnol.

[33] Zhang A, Zhuang X, Chen K, Huang S, Xu C, Fang B (2019). Adaptive evolution of *Clostridium butyricum* and scale-up for high-concentration 1,3-propanediol production. AIChE J.

[34] Szymanowska-Powałowska D, Kubiak P (2015). Effect of 1,3-propanediol, organic acids, and ethanol on growth and metabolism of *Clostridium butyricum* DSP1. Appl Microbiol Biotechnol.

[35] Serrano-Bermúdez LM, González Barrios AF, Maranas CD, Montoya D (2017). *Clostridium butyricum* maximizes growth while minimizing enzyme usage and ATP production: metabolic flux distribution of a strain cultured in glycerol. BMC Syst Biol.

[36] Zeng AP (1996). Pathway and kinetic analysis of 1,3-propanediol production from glycerol fermentation by *Clostridium butyricum*. Bioprocess Eng.

[37] Zeng AP, Deckwer WD (1995). A kinetic model for substrate and energy consumption of microbial growth under substrate-sufficient conditions. Biotechnol Progr.

[38] Boenigk R, Bowien S, Gottschalk G (1993). Fermentation of glycerol to 1,3-propanediol in continuous cultures of *Citrobacter freundii*. Appl Microbiol Biotechnol.

[39] Zhang C, Sharma S, Ma C, Zeng A (2022). Strain evolution and novel downstream processing with integrated catalysis enable highly efficient coproduction of 1,3-propanediol and organic acid esters from crude glycerol. Biotechnol Bioeng.

[40] Yang X, Choi HS, Lee JH, Lee SK, Han SO, Park C, Kim SW (2018). Improved production of 1,3-propanediol from biodiesel-derived crude glycerol by *Klebsiella pneumoniae* in fed-batch fermentation. Chem Eng J.

[41] Ji X, Huang H, Zhu J, Hu N, Li S (2009). Efficient 1,3-propanediol production by fed-batch culture of *Klebsiella pneumoniae*: the role of pH fluctuation. Appl Biochem Biotechnol.

[42] Merchuk JC, Asenjo JA (1995). The Monod equation and mass transfer. Biotechnol Bioeng.

[43] Silva JP, Almeida YB, Pinheiro IO, Knoelchelmann A, Silva JMF (2015). Multiplicity of steady states in a bioreactor during the production of 1,3-propanediol by *Clostridium butyricum*. Bioprocess Biosyst Eng.

[44] Luedeking R, Piret EL (1959). A kinetic study of the lactic acid fermentation. Batch process at controlled pH. J Biochem Microbiol Technol Eng.

[45] Xiu Z, Song B, Wang Z, Sun L, Feng E, Zeng A (2004). Optimization of dissimilation of glycerol to 1,3-propanediol by *Klebsiella pneumoniae* in one- and two-stage anaerobic cultures. Biochem Eng J.

[46] Wang H, Zhang N, Qiu T, Zhao J, He X, Chen B (2014). Optimization of a continuous fermentation process producing 1,3-propane diol with Hopf singularity and unstable operating points as constraints. Chem Eng Sci.

[47] Zhang A, Liu H, Huang S, Fu Y, Fang B (2018). Metabolic profiles analysis of 1,3-propanediol production process by *Clostridium butyricum* through repeated batch fermentation coupled with activated carbon adsorption. Biotechnol Bioeng.

[48] Wilkens E, Ringel AK, Hortig D, Willke T, Vorlop K (2012). High-level production of 1,3-propanediol from crude glycerol by *Clostridium butyricum* AKR102a. Appl Microbiol Biotechnol.

[49] Loureiro-Pinto M, Coca M, González-Benito G, Lucas S, García-Cubero MT (2017). Continuous bioproduction of 1,3-propanediol from biodiesel raw glycerol: operation with free and immobilized cells of *Clostridium butyricum* DSM 10702. Can J Chem Eng.

[50] Kaur G, Srivastava AK, Chand S (2013). Bioconversion of glycerol to 1,3-propanediol: a mathematical model-based nutrient feeding approach for high production using *Clostridium diolis*. Bioresour Technol.

[51] Supaporn P, Yeom SH (2018). Statistical optimization of 1,3-propanediol (1,3-PD) production from crude glycerol by considering four objectives: 1,3-PD concentration, yield, selectivity, and productivity. Appl Biochem Biotechnol.

[52] Pan D, Wang X, Wang J, Shi H, Wang G, Xiu Z (2022). Optimization and feedback control system of dilution rate for 1,3-propanediol in two-stage fermentation: a theoretical study. Biotechnol Progr.

[53] Kaur G, Srivastava AK, Chand S (2012). Mathematical modelling approach for concentration and productivity enhancement of 1,3-propanediol using *Clostridium diolis*. Biochem Eng J.

